# Clinical and Metabolic Predictors of Response to Focused Extracorporeal Shockwave Therapy in Rotator Cuff Tendinopathy: A Retrospective Cohort Study

**DOI:** 10.3390/medsci14010114

**Published:** 2026-02-27

**Authors:** Sveva Maria Nusca, Eleonora Latini, Gabriele Santilli, Gioia Beccarini, Valerio Bova, Flavia Santoboni, Valter Santilli, Giorgio Felzani, Fabrizio Perroni, Mariachiara Vulpiani, Davide Sisti, Mario Vetrano

**Affiliations:** 1 Physical Medicine and Rehabilitation Unit, Sant’Andrea Hospital, Sapienza University of Rome, 00189 Rome, Italy; 2 Department of Movement, Human and Health Sciences, University of Rome ‘Foro Italico’, 00135 Rome, Italy; 3 San Raffaele Sulmona, Viale dell’Agricoltura, 67039 Sulmona, Italy; 4 Department of Biomolecular Sciences, University of Urbino Carlo Bo, 61029 Urbino, Italy

**Keywords:** extracorporeal shockwave therapy, rotator cuff tendinopathy, predictive factors, supraspinatus, body mass index, rehabilitation

## Abstract

Background: Rotator cuff tendinopathy is a major cause of shoulder pain and disability. Focused extracorporeal shockwave therapy (ESWT) is an established conservative treatment option; however, the predictive factors influencing the treatment response remain poorly characterized. Objectives: To identify clinical, demographic, and metabolic predictors of pain reduction and functional improvement at four months following focused ESWT in patients with supraspinatus tendinopathy, with the goal of informing individualized treatment planning and early prognostic counseling. Methods: This retrospective cohort study analyzed patients with supraspinatus tendinopathy (calcific and non-calcific) treated with focused ESWT at a university rehabilitation center between June 2020 and December 2025. Outcomes were assessed at baseline and 4-month follow-up using the Visual Analog Scale (VAS), Roles and Maudsley, and Constant–Murley scores. Change score analysis with covariate adjustment and backward stepwise selection were performed to identify predictors of clinical improvement. Results: A total of 239 patients (97 males [40.6%], 142 females [59.4%]; mean age 60.2 ± 11.5 years; mean BMI 25.5 ± 4.0 kg/m^2^) were included, of whom 101 (42.3%) had calcific tendinopathy. Significant improvements were observed in all outcomes: VAS decreased from 6.50 ± 1.35 to 3.96 ± 2.09 (*p* < 0.001; Cohen’s d = 1.24), and Constant–Murley score increased from 60.38 ± 14.53 to 75.88 ± 15.52 (*p* < 0.001; Cohen’s d = 1.07). Patient-reported satisfaction (Roles and Maudsley score) showed a 91.2% success rate (excellent or good outcomes). Regression analysis identified baseline severity as the strongest predictor of improvement in all models. BMI emerged as a significant predictor of functional recovery (β = −0.95, *p* < 0.001 for Constant–Murley change), with each 1 kg/m^2^ increase associated with approximately 1-point less improvement. Conclusions: Baseline clinical severity and body mass index were consistent predictors of ESWT effectiveness in rotator cuff tendinopathy. A lower BMI was associated with greater functional improvement, highlighting a potentially modifiable factor for treatment optimization. These findings support personalized treatment planning and early prognostic counseling in clinical practices.

## 1. Introduction

Shoulder disorders are common musculoskeletal problems worldwide, with prevalence rates ranging from 10.8% to 55.2% in a 12-month reference period [1]. One of the most frequent causes is rotator cuff tendinopathy, an umbrella term encompassing conditions affecting the subacromial structures, including tendinitis, calcific tendinopathy, and impingement syndrome [2,3]. The supraspinatus tendon is the most commonly involved structure in these injuries. Tendinopathy involves complex pathological changes at the cellular and extracellular matrix levels, including collagen fiber disorganization, increased proteoglycan and glycosaminoglycan content, neovascularization, and aberrant tenocyte proliferation. The “continuum model” proposed by Cook and Purdam describes tendinopathy progression through three stages: reactive tendinopathy (short-term adaptation to acute overload), tendon disrepair (failed healing with matrix breakdown), and degenerative tendinopathy (cell death and matrix disorganization). This framework has important implications for treatment selection and prognosis, as therapeutic responsiveness may vary across disease stages [2,3,4].

Management is predominantly conservative, with exercise therapy being strongly recommended [5]. Other treatment options include manual therapy, injections, and physical modalities, although high-quality evidence remains limited [5,6,7,8]. Unfortunately, approximately 40% of patients fail to respond to conservative treatment [9], and more than half report persistent long-term symptoms [10].

The etiology is multifactorial, involving extrinsic factors (anatomical variations, biomechanical dysfunction, overuse) and intrinsic factors (tendon biology alterations, vascularity, and genetic predisposition) [11,12,13]. Multiple risk factors have been associated with rotator cuff disease, including age > 50 years [11,12,13,14], female sex [1,15], smoking [16], diabetes mellitus [17], hypertension, hyperlipidemia [17], thyroid disorders [18], and obesity [19,20]. These factors may influence both disease development and treatment responses.

Focused extracorporeal shockwave therapy (f-ESWT) is recognized as a valid and safe treatment for rotator cuff tendinopathy [21], with recent meta-analyses confirming its efficacy in both calcific and non-calcific forms [22,23,24,25]. Extracorporeal shockwave therapy delivers focused acoustic energy pulses to affected tissues, inducing mechanotransduction-mediated cellular responses. Proposed therapeutic mechanisms include stimulation of growth factors (VEGF, TGF-β, BMP-2), enhanced neovascularization, modulation of inflammatory mediators, and activation of tenocyte proliferation and matrix synthesis [21].

Previous research has established ESWT efficacy for rotator cuff tendinopathy, with recent meta-analyses confirming significant improvements in pain and function for both calcific and non-calcific subtypes [22,23]. While isolated studies have examined individual predictive factors such as symptom duration or lesion characteristics, comprehensive evaluation of clinical, anthropometric, and metabolic predictors remains lacking. This knowledge gap limits the ability to provide individualized prognosis and optimize patient selection.

The present study aimed to identify, through interpretable regression modeling, the key predictors of ESWT response in a large, heterogeneous cohort encompassing both calcific and non-calcific presentations.

## 2. Materials and Methods

### 2.1. Study Design and Population

This single-center retrospective cohort study analyzed consecutive patients treated with f-ESWT for supraspinatus tendinopathy at the Physical Medicine and Rehabilitation Unit of Sant’Andrea University Hospital, Rome, Italy, between June 2020 and December 2025. The study was approved by the Institutional Review Board of La Sapienza University (Prot. 0000/2024—Approval Date: 14 February 2024) and conducted in accordance with the Declaration of Helsinki. All the participants provided written informed consent. This study followed the STROBE guidelines for observational studies [26].

### 2.2. Participants

**Figure 1:**
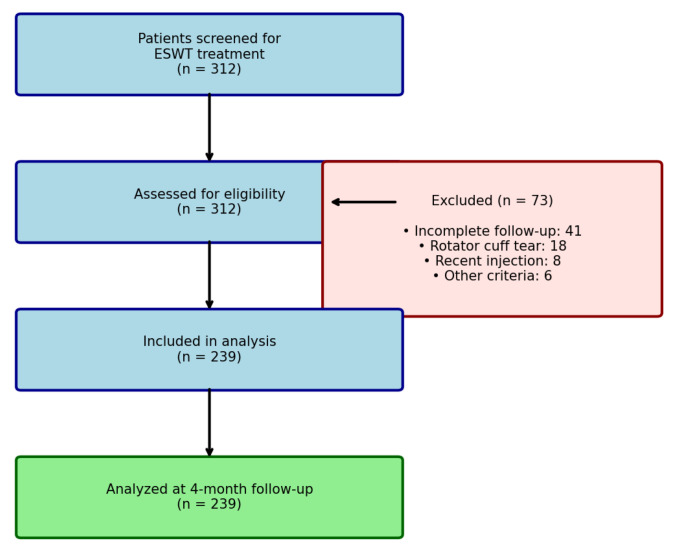
Patient Flow Diagram.

Eligible patients were adults with chronic supraspinatus tendinopathy (calcific or non-calcific) who were referred for f-ESWT. Diagnosis was established through clinical evaluation and multimodal imaging. The clinical diagnostic criteria included: (1) shoulder pain ≥3 months in duration; (2) pain exacerbation during overhead activities or at night; (3) positive findings on at least two of the following clinical tests: Jobe test (empty can), Neer impingement sign, Hawkins test, or painful arc between 60° and 120° of abduction. Imaging was performed using a standardized protocol. Tendon pathology was confirmed using ultrasonography and/or magnetic resonance imaging. The ultrasound criteria included tendon thickening, hypoechogenicity, loss of fibrillar pattern, and/or partial-thickness tears. The MRI criteria included increased signal intensity on T2-weighted or proton density sequences with changes in tendon morphology. Standard anteroposterior and outlet-view shoulder radiographs were obtained for all patients to confirm the presence or absence of calcifications within the rotator cuff. The inclusion criteria were as follows: (1) age ≥ 18 years; (2) clinical and imaging-confirmed supraspinatus tendinopathy; (3) symptom duration ≥ 3 months; and (4) complete baseline and follow-up assessments. The exclusion criteria were as follows: (1) previous ipsilateral shoulder surgery; (2) full-thickness rotator cuff tear; (3) adhesive capsulitis (primary or secondary); (4) corticosteroid injection or suprascapular nerve block within 6 weeks prior to f-ESWT; (5) active malignancy; (6) local infection; (7) pregnancy; (8) coagulopathy or anticoagulant therapy; and (9) cardiac pacemaker or implantable defibrillator. During the study period, 312 patients were screened for their eligibility. Seventy-three patients were excluded (41 with incomplete follow-up, 18 with rotator cuff tears on imaging, 8 with recent injections, and 6 with other exclusion criteria), yielding a final sample of 239 patients (Figure 1).

### 2.3. Intervention

All f-ESWT sessions were performed by two experienced physiatrists (>5 years of ESWT experience, >500 procedures each) using the Modulith SLK system (Storz Medical AG, Tägerwilen, Switzerland), an electromagnetic focused shockwave generator with integrated inline ultrasound guidance. Patient positioning: supine with the affected arm in neutral adduction, elbow flexed at 90°, and the forearm resting on the abdomen. Treatment parameters: The shockwave applicator was positioned under real-time ultrasound guidance to target the supraspinatus tendon insertion at the greater tuberosity of the humerus. Treatment was delivered without local anesthesia using the following standardized protocol: energy flux density 0.15 mJ/mm^2^ (low-to-medium energy); 2400 pulses per session; frequency 4 Hz; 3 treatments at weekly intervals. All patients received a standardized home exercise program consisting of pendulum exercises, passive and active-assisted range of motion, and progressive rotator cuff strengthening. Patients were instructed to perform exercises daily throughout the treatment and follow-up periods. No other physical therapy modalities were permitted during the study.

### 2.4. Rehabilitation Program

In addition to f-ESWT, all participants followed a standardized rehabilitation program specifically designed for rotator cuff-related shoulder pain. The rehabilitation program was initiated concomitantly with the first f-ESWT session and continued for a total duration of 16 weeks. Rehabilitation sessions were performed three times per week and supervised by an experienced physical therapist specialized in shoulder rehabilitation to ensure correct execution and appropriate progression of exercises; adherence was monitored through direct supervision and attendance records. Each session lasted approximately 45–60 min and began with a standardized warm-up consisting of low-intensity upper-limb ergometer activity and active shoulder mobility exercises within a pain-free range. The core component of the program consisted of progressive strengthening exercises targeting the rotator cuff and scapular stabilizers, including isometric shoulder abduction and external rotation, side-lying external rotation, prone horizontal abduction, and elastic resistance exercises for external and internal rotation with the arm at 0–45° of abduction. Scapular-focused exercises (such as prone Y and T raises and serratus anterior strengthening) were also included. Stretching exercises for the posterior shoulder capsule and pectoralis minor were performed at the end of each session [27,28,29]. Exercise intensity and difficulty were progressively increased based on individual tolerance and symptom response, following a pain-monitoring approach allowing mild discomfort without symptom exacerbation to avoid kinesiophobia [30,31,32,33]. All clinical outcome assessments were conducted on days separate from f-ESWT sessions to minimize the influence of acute treatment-related effects.

### 2.5. Outcome Measures

Clinical outcomes were assessed at two time points: baseline (T0, immediately before the first f-ESWT session) and follow-up (T1, four months after completion of the ESWT protocol). The 4-month follow-up interval was selected based on both clinical and biological rationales. Clinically, this timeframe represents a pragmatic decision point when treatment response can be meaningfully assessed and alternative strategies considered if improvement is insufficient. Biologically, ESWT-induced tissue regeneration—including neovascularization, collagen remodeling, and inflammatory resolution—typically requires 12–16 weeks to manifest as clinical improvement [21]. This interval also allows differentiation between sustained therapeutic effects and transient post-procedural responses. All assessments were performed by trained physiotherapists who were not involved in the treatment delivery. The primary endpoint was the Visual Analog Scale (VAS); it consists of a 100 mm horizontal line anchored by “no pain” on the left (score 0 mm) and “worst imaginable pain” on the right (score 100 mm). Participants were asked to mark the point that best represented the intensity of pain experienced during their most painful movement. The value was quantified by measuring, in millimeters, the distance from the left anchor to the patient’s mark [34]. Secondary outcome measures comprised: the Roles and Maudsley score (RM), a validated four-point ordinal scale assessing patient-reported treatment outcome: 1 = excellent (no pain, full movement, and activity); 2 = good (occasional discomfort, full function); 3 = acceptable (some discomfort after prolonged activity); 4 = poor (unchanged or worse symptoms). Treatment success was defined as a score of 1 or 2 at follow-up [35]; and Constant–Murley score (CMS): a validated 100-point composite measure of shoulder function comprising pain (15 points), activities of daily living (20 points), range of motion (40 points), and strength (25 points). Higher scores indicate better functions [36,37]. For the VAS and Constant–Murley score, change scores were calculated as the difference between the baseline and follow-up values. For the VAS, improvement was represented by positive change scores (T0 minus T1). For the Constant–Murley score, improvement was represented by positive change scores (T1 minus T0).

### 2.6. Predictor Variables

Candidate predictor variables were selected a priori based on existing literature on rotator cuff tendinopathy, tendon healing mechanisms, and response to f-ESWT. Data were extracted from electronic medical records and included demographic, anthropometric, lifestyle, occupational, and clinical factors.

Demographic variables included age (years, continuous) and sex (male/female). Anthropometric variables included body mass index (BMI, kg/m^2^, continuous), calculated from measured height and weight at baseline. BMI was included given the established association between adiposity, chronic low-grade inflammation, and impaired tendon healing.

Lifestyle and occupational factors comprised current smoking status (yes/no). Participants were classified as smokers if they reported smoking cigarettes regularly (i.e., at least daily) [38]; regular sports participation (yes/no), was defined based on self-reported frequency of sport involvement, participants reporting sports activity at least once per week were classified as engaging in regular sports participation, whereas those reporting no or infrequent participation (less than once per week) were classified as non-regular participants [39]; participation in overhead sports involving repetitive shoulder use (yes/no) and occupational shoulder risk (yes/no), defined as work requiring sustained arm elevation above 90° or repetitive overhead movements [40].

Medical comorbidities were recorded as physician-diagnosed conditions documented in the medical records and included diabetes mellitus, hypercholesterolemia, arterial hypertension, thyroid disease, rheumatic disease, and previous ipsilateral shoulder trauma. Disease-specific characteristics included the presence or absence of supraspinatus tendon calcification on baseline radiographic assessment.

### 2.7. Statistical Analysis

All statistical analyses were performed using R statistical software version 4.3.0 (R Foundation for Statistical Computing, Vienna, Austria). As this was a retrospective exploratory cohort study, formal a priori sample size calculation was not performed. However, the final sample of 239 patients substantially exceeded the recommended minimum of 10–15 subjects per predictor variable for stable regression coefficient estimation [41]. Post hoc power analysis for the Constant–Murley model (observed f^2^ = 0.38, *n* = 239, 6 predictors, α = 0.05) indicated statistical power exceeding 0.99 for detecting significant predictors.

Descriptive statistics were used to summarize baseline characteristics. Continuous variables are presented as mean ± standard deviation, whereas categorical variables are reported as absolute frequencies and percentages. Baseline characteristics were compared between sexes using independent *t*-tests for continuous variables and chi-squared tests for categorical variables.

To identify predictors of clinical improvement at 4 months, two separate multivariable linear regression models were constructed, with change scores as dependent variables: (1) change in VAS pain score and (2) change in Constant–Murley score. For both outcomes, change scores were calculated as the difference between baseline and follow-up values, with positive values indicating clinical improvement. Each model included the corresponding baseline score as a covariate to account for baseline severity and to reduce the effect of regression to the mean [42].

All candidate predictors were initially entered into the full models. Variable selection was performed using backward stepwise elimination based on the Akaike Information Criterion (AIC), with the aim of balancing model fit and parsimony [43]. Given the exploratory nature of this analysis, the identified associations should be interpreted as preliminary and hypothesis-generating rather than confirmatory. We acknowledge that stepwise procedures may produce unstable coefficient estimates and that the identified variables cannot be considered definitive predictors without external validation in independent cohorts [44].

Multicollinearity was assessed using variance inflation factors (VIF), with values below 3 considered acceptable. Model performance was evaluated using the adjusted coefficient of determination (adjusted R^2^) [45], which provides an estimate of explained variance but does not imply causal inference or definitive predictive capability. Statistical significance was set at a two-tailed α level of 0.05.

## 3. Results

### 3.1. Patient Characteristics

The final cohort comprised 239 patients: 97 men (40.6%) and 142 women (59.4%), with a mean age of 60.2 ± 11.5 years. Males were significantly older than females (62.1 ± 12.4 vs. 59.0 ± 10.7 years; *p* = 0.036). Mean BMI was 25.5 ± 4.0 kg/m^2^, with males showing higher values than females (26.4 ± 3.5 vs. 24.8 ± 4.3 kg/m^2^; *p* = 0.005). Calcification was observed in 101 patients (42.3%). Significant sex differences were observed for several comorbidities and risk factors: thyroid disease was more prevalent in females (28.9% vs. 6.2%; *p* < 0.001), while overweight/obesity (BMI ≥ 25 kg/m^2^: 66.0% vs. 38.0%; *p* < 0.001), smoking (39.2% vs. 24.6%; *p* = 0.024), overhead sports participation (28.9% vs. 9.9%; *p* < 0.001), and hypertension (46.4% vs. 30.3%; *p* = 0.016) were more common in males. Table 1 presents the complete baseline characteristics.

### 3.2. Clinical Outcomes

Statistically significant and clinically meaningful improvements were observed across all outcome measures at the 4-month follow-up (Table 2). Pain intensity (VAS) decreased from 6.50 ± 1.35 at baseline to 3.96 ± 2.09 at follow-up, representing a mean reduction of 2.55 points (95% CI: 2.29–2.81; *p* < 0.001), corresponding to a 39% decrease from the baseline. The effect size was large (Cohen’s d = 1.24). Patient-reported treatment satisfaction, assessed using the Roles and Maudsley score at the 4-month follow-up, demonstrated favorable outcomes. Most patients reported excellent (*n* = 147, 61.5%) or good (*n* = 71, 29.7%) results, yielding an overall success rate of 91.2%. Acceptable outcomes were reported by 20 patients (8.4%), whereas only one patient (0.4%) reported a poor result. Shoulder function, as measured by the Constant–Murley score, increased from 60.38 ± 14.53 to 75.88 ± 15.52, reflecting a mean improvement of 15.50 points (95% CI: 13.67–17.34; *p* < 0.001; Cohen’s d = 1.07).

### 3.3. Predictors of Treatment Response

**Figure 2:**
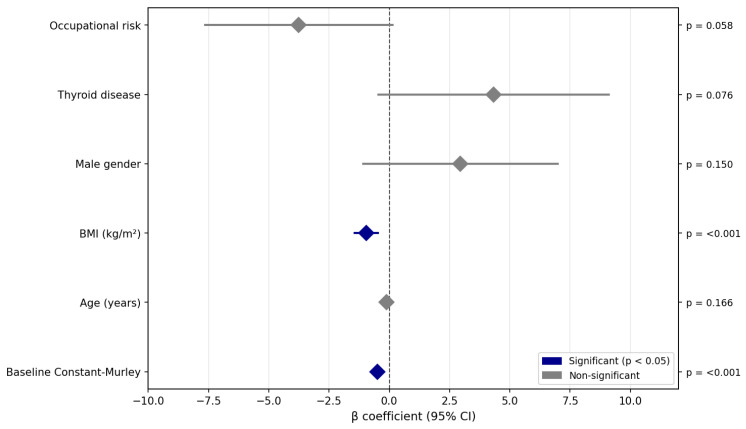
Predictors of Constant–Murley Score Change at 4 Months.

Two regression models were constructed to identify the predictors of clinical improvement at 4 months (Table 3). Both models were statistically significant (*p* < 0.001). Pain Reduction (Delta VAS): The model explained 12.2% of the variance (adjusted R^2^ = 0.122). A higher baseline VAS score was the strongest predictor of pain reduction (β = 0.57; 95% CI: 0.37–0.77; *p* < 0.001), indicating a greater absolute improvement in patients with more severe initial pain. A higher baseline Constant–Murley score was also associated with greater pain reduction (β = 0.025; 95% CI: 0.01–0.04; *p* = 0.010). The absence of calcification showed a trend toward greater VAS improvement (β = −0.43; *p* = 0.105). Shoulder Function (Constant–Murley): The model explained 27.5% of the variance (adjusted R^2^ = 0.275). Two variables emerged as significant predictors: baseline Constant–Murley score (β = −0.50; 95% CI: −0.63 to −0.38; *p* < 0.001) and BMI (β = −0.95; 95% CI: −1.43 to −0.47; *p* < 0.001) (Figure 2). The negative coefficient for BMI indicates that each 1 kg/m^2^ increase was associated with approximately 1-point less improvement in the Constant–Murley score at 4 months. Male sex showed a trend toward better functional outcomes (β = 2.95; *p* = 0.150), and the presence of thyroid disease showed a non-significant positive association (β = 4.33; *p* = 0.076).

Notably, common metabolic comorbidities, including diabetes mellitus, hypercholesterolemia, and hypertension, were not retained in any final model, indicating no significant independent association with treatment outcomes at 4 months.

### 3.4. Safety

No serious adverse events related to f-ESWT were observed during the study. Transient local discomfort during treatment was reported by some patients but did not require discontinuation of treatment. All patients completed the prescribed three sessions.

## 4. Discussion

This retrospective cohort study including 239 patients with supraspinatus tendinopathy, encompassing both calcific and non-calcific subtypes, identified baseline functional severity and body mass index (BMI) as significant early prognostic factors associated with clinical improvement following focused extracorporeal shockwave therapy at 4 months. Improvement in the Constant–Murley score was inversely associated with BMI, while baseline symptom severity consistently emerged as the strongest independent factor across outcome models. In contrast, common metabolic comorbidities, including diabetes mellitus, hypertension, and hypercholesterolemia, were not significantly associated with treatment outcomes. Overall patient-reported satisfaction was high, with 91.2% of patients reporting excellent or good postoperative outcomes.

The inverse association between BMI and functional improvement remained statistically significant after adjustment for confounders (β = −0.95; 95% CI: −1.43 to −0.47; *p* < 0.001). Each 1 kg/m^2^ increase in BMI corresponded to an approximate 1-point reduction in Constant–Murley score improvement at 4 months. Considering that the minimal clinically important difference for the Constant–Murley score has been estimated at 10–17 points [46], the magnitude of this association suggests a potentially relevant attenuation of treatment response in patients with higher BMI.

This association aligns with the growing evidence linking adiposity to impaired tendon healing through chronic low-grade inflammation and oxidative stress [47,48,49]. Adipose tissue secretes pro-inflammatory cytokines, including interleukin-6 and tumor necrosis factor-α, which may disrupt tendon matrix homeostasis and attenuate the regenerative effects of f-ESWT [47]. Elevated oxidative stress in obese individuals may further compromise tenocyte viability and collagen synthesis, thereby diminishing the mechanotransductive response to shockwave stimulation [49]. From a clinical perspective, BMI represents a modifiable factor, unlike age or genetic predisposition, suggesting that weight management strategies could potentially enhance f-ESWT responsiveness. Clinicians should consider integrating nutritional counseling or supervised exercise programs into rehabilitation plans for overweight patients, although prospective interventional studies are needed to confirm whether BMI reduction improves f-ESWT outcomes.

Baseline severity emerged as the strongest predictor of improvement across both outcome measures, with higher baseline pain and functional limitation associated with greater absolute improvement following treatment, consistent with previous prognostic evidence in rotator cuff-related shoulder pain [50,51]. While regression to the mean may partially explain this phenomenon—patients at extreme values tend to move toward the population mean upon reassessment—the consistency and magnitude of this association across models support its genuine clinical utility. Patients presenting with more severe functional limitations may represent a subgroup with greater potential for measurable improvement, supporting early f-ESWT intervention in moderate-to-severe cases. However, clinicians should frame expectations appropriately: while severely affected patients may achieve larger absolute gains, they may not reach the same absolute functional level as patients with milder baseline impairments. The baseline score thus serves dual purposes—as an indicator of disease burden and as a predictor of therapeutic responsiveness—enabling more informed discussions about realistic outcome expectations [52,53,54]. Male sex showed a trend toward greater Constant–Murley score improvement (β = 2.95; *p* = 0.150), although this did not reach statistical significance. This observation is consistent with the emerging evidence of hormonal influences on tendon biology. Estrogen modulates collagen synthesis, tenocyte proliferation, and inflammatory responses in tendons [55], and estrogen receptor expression has been observed in the rotator cuff [56]. Lower estrogen levels in males may confer greater tendon stiffness and resilience, potentially enhancing the response to mechanical stimulation from f-ESWT. Additionally, the higher prevalence of thyroid disease observed in females in our cohort (28.9% vs. 6.2%) may represent a confounding factor, as thyroid dysfunction has been associated with altered tendon metabolism [18]. Given the trend-level significance, these findings should be interpreted cautiously and warrant investigation in adequately powered prospective studies in the future.

In the treatment of musculoskeletal pain, increasing attention has been directed toward combined therapeutic strategies, including physical physiotherapeutic modalities and approaches that integrate these with active rehabilitation, with the aim of addressing both pain reduction and the restoration of tissue load capacity [57,58,59,60,61,62]. In this context, combined therapeutic strategies that integrate f-ESWT and active rehabilitation have demonstrated beneficial effects on pain and function in patients with rotator cuff–related conditions [63]. Previous studies have consistently shown that active exercise therapy is superior to passive interventions in improving pain, shoulder function, and long-term outcomes in rotator cuff disorders, highlighting the central role of load-based rehabilitation in tendon recovery [64,65]. Building on this evidence, the present study adopted a combined approach integrating f-ESWT within a structured exercise-based rehabilitation program to support both symptom modulation and functional recovery.

With regard to the factors that may predispose to clinical success, the recent scientific literature has not extensively investigated them in the context of rotator cuff tendinopathy. In particular, there is a relative paucity of studies specifically aimed at identifying prognostic indicators associated with favorable outcomes following conservative management [8,51]. Conversely, a substantial body of evidence is available concerning predictors of successful outcomes after surgical treatment of rotator cuff pathology, especially in cases of full-thickness tendon tears, where variables such as patient age, tear size, tendon retraction, fatty degeneration, and comorbidities have been widely explored [66,67,68].

The 4-month prognostic window evaluated in this study has substantial practical relevance. Patients seeking treatment for shoulder pain expect improvement within a clinically meaningful timeframe, and the ability to predict likely responders versus non-responders at an early stage enables several clinical applications. First, prognostic information can inform shared decision-making, helping patients understand realistic outcome expectations based on their individual profiles. Second, the early identification of patients unlikely to respond favorably may prompt the timely consideration of alternative or adjunctive interventions, such as ultrasound-guided injections or surgical consultation. Third, the recognition of modifiable prognostic factors, particularly BMI, opens up opportunities for optimization strategies prior to or concurrent with f-ESWT.

The use of widely available clinical variables (BMI and baseline scores) enhances the feasibility of implementing prognostic assessment in routine practice without requiring specialized testing. While the explanatory power of our models was moderate—higher for Constant–Murley (R^2^ = 0.28) and lower for VAS pain (R^2^ = 0.12)—even modest predictive accuracy provides clinically useful information when combined with clinical judgment.

## 5. Limitations

This study offers several limitations that must be acknowledged. The retrospective design of this study introduced potential selection and information biases. The loss of 41 patients to follow-up (13.1%) may introduce attrition bias, although this rate was within the acceptable limits for rehabilitation research. The single-center setting limits geographic and practice variations. The 4-month follow-up precluded conclusions about the long-term durability of f-ESWT effects or late-emerging predictive factors. Finally, backward stepwise variable selection, while pragmatic for exploratory analysis, may yield unstable coefficient estimates; accordingly, the identified predictors should be considered hypothesis-generating and require validation in independent, prospective cohorts.

## 6. Conclusions

In this retrospective cohort of 239 patients with rotator cuff tendinopathy, baseline functional severity and BMI emerged as significant predictors of clinical response to focused ESWT at 4 months. The inverse association between BMI and functional improvement may reflect adiposity-related impairment of tendon healing through inflammatory and metabolic pathways. Common metabolic comorbidities did not significantly influence short-term outcomes. These hypothesis-generating findings suggest that BMI may represent a modifiable factor for treatment optimization. Future prospective studies should validate these predictors, explore underlying mechanisms through biomarker assessment, and evaluate whether weight management interventions enhance ESWT effectiveness.

## Figures and Tables

**Table 1:** Baseline Characteristics of the Study Population.

Variable	Total (*n* = 239)	Male (*n* = 97)	Female (*n* = 142)	*p*-Value
Demographics				
Age, years	60.2 ± 11.5	62.1 ± 12.4	59.0 ± 10.7	0.036
BMI, kg/m^2^	25.5 ± 4.0	26.4 ± 3.5	24.8 ± 4.3	0.005
Comorbidities				
BMI ≥ 25 kg/m^2^	118 (49.4%)	64 (66.0%)	54 (38.0%)	<0.001
Diabetes mellitus	41 (17.2%)	18 (18.6%)	23 (16.2%)	0.764
Hypertension	88 (36.8%)	45 (46.4%)	43 (30.3%)	0.016
Hypercholesterolemia	73 (30.5%)	36 (37.1%)	37 (26.1%)	0.093
Thyroid disease	47 (19.7%)	6 (6.2%)	41 (28.9%)	<0.001
Rheumatic disease	14 (5.9%)	5 (5.2%)	9 (6.3%)	0.919
Lifestyle factors				
Current smoking	73 (30.5%)	38 (39.2%)	35 (24.6%)	0.024
Sports participation	98 (41.0%)	43 (44.3%)	55 (38.7%)	0.465
Overhead sports	42 (17.6%)	28 (28.9%)	14 (9.9%)	<0.001
Occupational risk	68 (28.5%)	23 (23.7%)	45 (31.7%)	0.232
Clinical characteristics				
Previous trauma	17 (7.1%)	8 (8.2%)	9 (6.3%)	0.758
Calcification present	101 (42.3%)	43 (44.3%)	58 (40.8%)	0.688

Values are mean ± SD or *n* (%). BMI = body mass index. Bold *p*-values indicate statistical significance (*p* < 0.05).

**Table 2:** Clinical Outcomes at Baseline and 4-Month Follow-up.

Panel A. Continuous Outcomes						
Outcome	Baseline (T0)	Follow-up (T1)	Change	95% CI	*p*-Value	Cohen’s d
VAS (0–10)	6.50 ± 1.35	3.96 ± 2.09	2.55 ± 2.05	2.29–2.81	<0.001	1.24
Constant–Murley (0–100)	60.38 ± 14.53	75.88 ± 15.52	15.50 ± 14.43	13.67–17.34	<0.001	1.07
Panel B. Roles and Maudsley Score (Ordinal)						
Category		Baseline *n* (%)		Follow-up *n* (%)		
1—Excellent		39 (16.3%)		147 (61.5%)		
2—Good		114 (47.7%)		71 (29.7%)		
3—Acceptable		75 (31.4%)		20 (8.4%)		
4—Poor		11 (4.6%)		1 (0.4%)		
Success rate (score 1–2)		153 (64.0%)		218 (91.2%)		

Values are mean ± SD. VAS = Visual Analog Scale; CI = confidence interval.

**Table 3:** Predictors of Clinical Improvement: Final Regression Models.

Predictor	β	SE	95% CI	*p*-Value
Model 1: Delta VAS				
Baseline VAS	0.571	0.101	0.37–0.77	<0.001
Baseline Constant–Murley	0.025	0.010	0.01–0.04	0.010
Male gender	0.182	0.265	−0.34–0.71	0.493
Smoking	−0.419	0.279	−0.97–0.13	0.134
Occupational risk	−0.531	0.285	−1.09–0.03	0.064
Calcification	−0.428	0.263	−0.95–0.09	0.105
Model 2: Delta Constant–Murley				
Baseline Constant–Murley	−0.504	0.064	−0.63–−0.38	<0.001
Age (years)	−0.113	0.081	−0.27–0.05	0.166
BMI (kg/m^2^)	−0.949	0.244	−1.43–−0.47	<0.001
Male gender	2.951	2.044	−1.08–6.98	0.150
Thyroid disease	4.328	2.424	−0.45–9.11	0.076
Occupational risk	−3.756	1.972	−7.64–0.13	0.058

β = unstandardized regression coefficient; SE = standard error; CI = confidence interval.

## Data Availability

The data presented in this study are available on request from the corresponding author. (The datasets used, and data analyzed during the current study will be made available upon reasonable request to the corresponding author.)
